# Tau aggregation and its interplay with amyloid-β

**DOI:** 10.1007/s00401-014-1371-2

**Published:** 2014-12-10

**Authors:** Rebecca M. Nisbet, Juan-Carlos Polanco, Lars M. Ittner, Jürgen Götz

**Affiliations:** 1 Clem Jones Centre for Ageing Dementia Research, Queensland Brain Institute, The University of Queensland, Brisbane, Australia; 2 Dementia Research Unit, Wallace Wurth Building, The University of New South Wales, Sydney, Australia

**Keywords:** Alzheimer’s disease, Amyloid-β (Aβ), Frontotemporal dementia, Tau, Immunotherapy, scFvs (single-chain variable antibody fragments), Neurotoxicity

## Abstract

Neurofibrillary tangles and amyloid plaques constitute the hallmark brain lesions of Alzheimer’s disease (AD) patients. Tangles are composed of fibrillar aggregates of the microtubule-associated protein tau, and plaques comprise fibrillar forms of a proteolytic cleavage product, amyloid-β (Aβ). Although plaques and tangles are the end-stage lesions in AD, small oligomers of Aβ and tau are now receiving increased attention as they are shown to correlate best with neurotoxicity. One key question of debate, however, is which of these pathologies appears first and hence is upstream in the pathocascade. Studies suggest that there is an intense crosstalk between the two molecules and, based on work in animal models, there is increasing evidence that Aβ, at least in part, exerts its toxicity via tau, with the Src kinase Fyn playing a crucial role in this process. In other experimental paradigms, Aβ and tau have been found to exert both separate and synergistic modes of toxicity. The challenge, however, is to integrate these different scenarios into a coherent picture. Furthermore, the ability of therapeutic interventions targeting just one of these molecules, to successfully neutralize the toxicity of the other, needs to be ascertained to improve current therapeutic strategies, such as immunotherapy, for the treatment of AD. Although this article is not intended to provide a comprehensive review of the currently pursued therapeutic strategies, we will discuss what has been achieved by immunotherapy and, in particular, how the inherent limitations of this approach can possibly be overcome by novel strategies that involve single-chain antibodies.

## Alzheimer’s disease

Alzheimer’s disease (AD) is a progressive neurodegenerative disease that is characterized by the formation of insoluble protein aggregates as well as the loss of neurons and synapses. This causes a progressive decline in memory and other cognitive functions that ultimately results in dementia; however, the processes leading to protein aggregation and neurodegeneration are only incompletely understood [5]. AD was first described in 1907 by Alois Alzheimer who reported two pathological hallmarks in the brain: amyloid plaques in the extracellular milieu and neurofibrillary tangles (NFTs) within neurons. It was not until eight decades later that the major proteinaceous components of these lesions were identified. Amyloid plaques consist primarily of aggregates of the amyloid-β peptide (Aβ) [28], whereas the main constituent of neurofibrillary tangles is the protein tau in a hyperphosphorylated form [34]. To this day, Aβ and tau remain the major therapeutic targets for the treatment of AD.

According to the 2010 World Alzheimer Report, it is estimated that there are presently 35.6 million people living with AD and related disorders, and this figure is expected to increase to 115 million by 2050 due to an increasingly aged population. As current treatments are purely for modest symptomatic relief, there is an urgent need for an effective therapeutic for the disease.

## The amyloid-β peptide

**Fig. 1:**
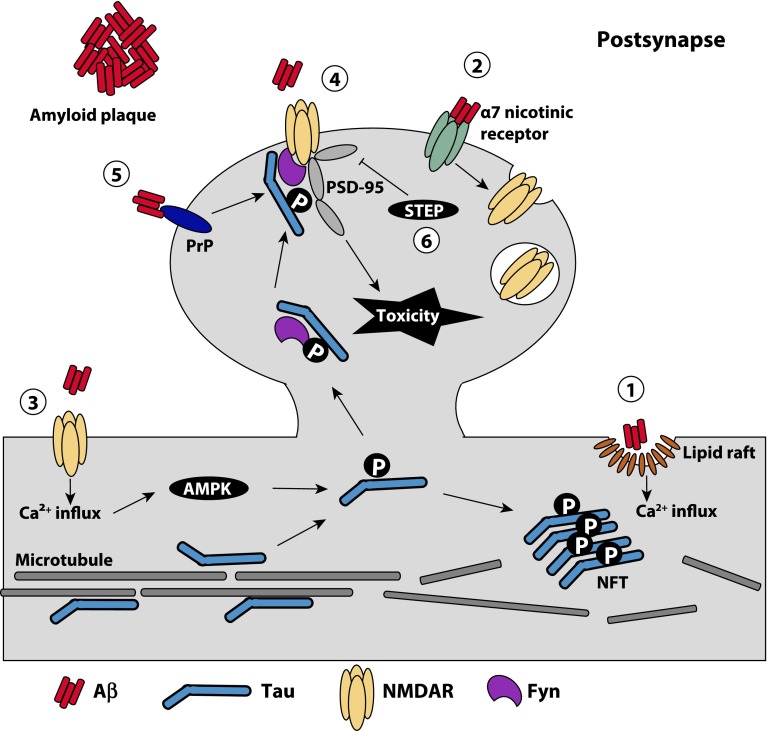
Proposed mechanisms underlying the toxic interplay between Aβ and tau at the synapse. Aβ oligomers have been demonstrated to exert their toxicity at the synapse through a number of mechanisms: (*1*) Binding of Aβ to the plasma membrane forms pores in the membrane, which may be facilitated by lipid rafts, resulting in calcium influx into the cell and the downstream activation of kinases implicated in tau phosphorylation. (*2*) Aβ can mediate the internalization of synaptic NMDARs indirectly through the binding of α7 nicotinic receptors. This results in a reduction of NMDARs at the synapse and causes synaptic spine shrinkage and retraction. (*3*) Aβ can mediate the activation of extrasynaptic NMDARs, which also induces a calcium influx into the neuron and in turn activates kinases such as AMPK. Activated kinases can phosphorylate dendritic tau which not only causes tau to detach from microtubules and aggregate into NFTs, but also enhances its binding to Fyn and results in the migration of tau and Fyn into the dendritic spine. (*4*) Within the dendritic spine, Fyn phosphorylates NMDARs and thereby mediates their interaction with PSD-95—an interaction required for Aβ toxicity. (*5*) Binding of Aβ to PrP^c^ also can activate Fyn to phosphorylate the NMDARs. (*6*) As the disease progresses, Aβ can activate the Fyn phosphatase STEP, which inactivates Fyn, resulting in the loss of synapses and dendritic spine collapse

Sequential cleavage of the amyloid precursor protein (APP) by β- and γ-secretase results in the generation of a range of Aβ peptides from 39 to 43 amino acid residues in length, although Aβ_1–__40_ and Aβ_1–__42_ are the predominant species in vivo. The hydrophobic nature of the peptides, particularly Aβ_1–__42_ and Aβ_1–__43_, allows them to self-aggregate and form a myriad of species from dimers to small molecular weight oligomers, to protofibrils, to fibrils, ultimately leading to their deposition as amyloid plaques **(**Fig. 1 **)**. Furthermore, Aβ peptides can also undergo pyroglutamate modification at amino acid position three (Aβ3(pE)) [63]; this increases the stability, aggregation propensity and neurotoxicity compared to full-length, unmodified Aβ. Mutations in the genes encoding *APP* and the γ-secretase components, *PSEN1* and *PSEN2*, lead to rare, early-onset familial forms of AD (FAD) by increasing the overall production of Aβ or shifting γ-secretase cleavage to produce more of the amyloidogenic Aβ_1–__42_. The mechanism by which excessive Aβ accumulation occurs in sporadic AD remains unclear. Reduced Aβ clearance or small increases in Aβ production over a long period of time are potential mechanisms that result in the accumulation of Aβ in the brain [61].

There is considerable debate regarding which Aβ species is the most neurotoxic, with increasing evidence suggesting that it is the small molecular weight oligomers that correlate best with disease [8, 52, 62, 67]. Consistent with this notion, secreted Aβ oligomers and protofibrils have been demonstrated to exert their toxicity at the synapse by binding to the cell surface, disrupting functional receptors and causing synaptic dysfunction **(**Fig. 1). The aggregation of Aβ at the cell surface is facilitated by membrane microdomains, termed lipid rafts, which are enriched in cholesterol and sphingolipids [37, 46]. Furthermore, the binding and disruption of functional receptors such as *N*-methyl-d-aspartate receptors (NMDARs) may also be lipid raft dependent [97]. Despite a lack of evidence for a direct binding of Aβ to NMDARs, Aβ negatively regulates the number of NMDARs at postsynaptic sites, thereby disrupting the balance between synaptic and extrasynaptic NMDAR activity [76]. Exposure of neurons to Aβ oligomers has been demonstrated to enhance NMDAR internalization and decrease synaptic expression [92]. This results in a decreased influx of Ca^2+^ into dendritic spines and causes synaptic spine shrinkage and retraction [90] (Fig. 1). A direct interaction between Aβ and a number of cell surface receptors, including the cellular prion protein (PrP^C^) [49], the scavenger receptor for glycation end products (RAGE) and the α7-nicotinergic (α7) receptor, has also been demonstrated [103, 104]. Binding of Aβ to the α7 receptor mediates the internalization of NMDARs, further shifting the balance towards extrasynaptic NMDAR signaling (Fig. 1). Furthermore, binding of fibrillar Aβ oligomers to PrP^C^ activates the Src kinase Fyn which increases phosphorylation of the NMDAR subunit NR2b, facilitates recruitment of the postsynaptic density (95 kda) protein (PSD-95), and eventually results in the loss of surface NMDARs [100] (Fig. 1). Recently, Aβ oligomers were also observed to induce apoptosis by activating transcription factor 4 (ATF4), which is translated and travels back to the cell body to initiate death signals [4]. It remains unclear, however, how the different interactions of Aβ are integrated in causing neurodegeneration.

## The microtubule-associated protein tau

Tau is a microtubule-associated protein (MAP) that is encoded by the *MAPT* gene. The protein contains an amino-terminal projection domain, a proline-rich region, a carboxy-terminal domain with microtubule-binding repeats, and a short tail sequence. Tau has been reported to interact with many proteins, serving important scaffolding functions [16]. In particular, it acts in concert with heterodimers of α- and β-tubulin to assemble microtubules and regulate motor-driven axonal transport. In the adult human brain, tau exists as six isoforms, which have either three or four microtubule-binding domains, which result from alternative splicing of exons two, three and ten of the *MAPT* gene; in contrast, the adult mouse brain only expresses the three isoforms with four microtubule-binding domains [57]. Tau is enriched in neurons where in mature neurons it is largely found in the axon, and is present to a small extent in the dendrites. The localization of tau in the dendritic compartments, including the spine, is tightly regulated and has a role in targeting Fyn [44].

The distinguishing factor that separates normal tau from that observed in patients with AD is its hyperphosphorylation. The longest isoform of human tau contains 80 serine and threonine residues and five tyrosine residues, all of which can potentially be phosphorylated. In the disease state, tau is hyperphosphorylated to a stoichiometry at least three times higher than that in the normal brain, with some sites phosphorylated to a higher degree than under physiological conditions and other sites de novo. It is not entirely clear, however, whether phosphorylation of tau at specific sites results in the pathogenicity observed in AD or whether it only requires a certain overall level of phosphorylation, as work in fly models would suggest [96]. Nevertheless, hyperphosphorylation of tau negatively regulates the binding of tau to microtubules, as a result of which microtubule stabilization and axonal transport are compromised. Hyperphosphorylation also increases the capacity of tau to self-assemble and form aggregates from oligomers to fibrils, eventually leading to its deposition as NFTs (Fig. 1). In addition, hyperphosphorylated tau has been shown to interfere with neuronal function, causing reduced mitochondrial respiration, altered mitochondrial dynamics and impaired axonal transport [26]. Tau pathology progresses through distinct neural networks and in AD, NFTs are prominent early in entorhinal cortex and later appear in anatomically connected brain regions [15]. This is thought to occur, in part, through the extracellular release of tau and recently it was shown that increasing neuronal activity increases the steady-state levels of extracellular tau in vivo [107]. Furthermore, it has been demonstrated that tau aggregates can have a seeding effect whereby extracellular tau can be internalized and upon internalization, these seeds can induce the fibrillization of endogenous tau [36]. This seeding can be detected prior to detection of tau pathology using histopathological markers, suggesting a role for tau seeding in neurodegeneration [40].

It is important to note that neurofibrillary lesions are also abundant in other neurodegenerative diseases, such as Pick’s disease, progressive supranuclear palsy, corticobasal degeneration, argyrophilic grain disease, and frontotemporal dementia with parkinsonism linked to chromosome 17 (FTDP-17), the latter caused by mutations in the *MAPT* gene. These diseases occur in the absence of overt Aβ deposition and demonstrate that tau dysfunction by itself can drive neurodegeneration [32].

## Synergistic roles of Aβ and tau in AD

Aβ and tau do not act in isolation, rather there is significant crosstalk between these two molecules [60, 86, 87]. Interestingly, whereas tau has been placed downstream of Aβ in a pathocascade, providing support for the amyloid cascade hypothesis [30, 55], a reduction in endogenous tau levels in APP transgenic mice was found to reverse memory impairment, reduce susceptibility to experimentally induced excitotoxic seizures, and decrease early mortality, without altering Aβ levels or plaque load [81]. This evidence of a crucial role of tau in synaptotoxicity is supported by the observation that hyperphosphorylated tau accumulates in dendritic spines of cultured CA3 hippocampal neurons [41, 112], and the finding that crossing human APP transgenic mice with human tau transgenic mice causes an enhanced aggregation of tau with concomitant dendritic spine loss, and accelerates cognitive impairment [20]. In pyramidal neurons of cortical layer V, however, hyperphosphorylated tau was not detected in the dendritic spines, even in neurons where the somatodendritic compartment was nearly completely filled with filamentous hyperphosphorylated tau, suggesting brain region-specific differences in this localization [38]. This difference may be explained by the fact that while dendritic spines of cortical pyramidal neurons have an actin-based cytoskeleton, the CA3 hippocampal neurons occasionally contain microtubules [22, 94].

As previously discussed, under physiological conditions tau has a critical role in targeting Fyn to the spine where it phosphorylates the NMDAR subunit NR2b, which recruits PSD-95 into a protein complex to mediate excitotoxicity. In the presence of elevated levels of Aβ (such as in APP mutant mice), NMDARs are overactivated, whether by direct or indirect binding, causing downstream toxicity. In a situation involving elevated levels of both Aβ and phosphorylated tau, more Fyn is targeted to the spine, augmenting the toxic effects of Aβ [44] (Fig. 1). For example, crossing APP mutant mice with transgenic mice carrying the P301L tau mutation, which is found in frontotemporal dementia, results in 100 % lethality by 4 months of age [43].

The binding of Fyn to tau is mediated by two domains, the SH2 domain that binds to tau via phosphorylated Tyr18, and the SH3 domain that binds to a series of PXXP motifs located in the amino-terminal projection domain of tau [50, 51]. Exclusion of Fyn from the dendrite in transgenic mice that either express only the amino-terminal projection domain of tau (Δtau) or that lack tau completely (tau^−/−^ mice) uncouples excitotoxicity in Aβ-depositing mice, strongly suggesting a dendritic role of tau in neuronal toxicity [44]. As these mice show reduced levels of postsynaptic Fyn and reduced phosphorylation of its substrate NR2b, which facilitates the interaction of NMDARs with PSD-95, it is believed that tau stabilizes this complex and enhances Aβ-induced toxicity at the synapse through NMDARs [44] (Fig. 1). Furthermore, increased intracellular calcium levels induced by Aβ-mediated over-excitation of extrasynaptic NMDARs has been demonstrated to activate the kinases AMPK and PAR-1/MARK, which in turn induce the phosphorylation of tau. In the presence of Aβ oligomers, AMPK has been demonstrated to cause phosphorylation of tau at Ser422 [59], an epitope that is only phosphorylated late in disease [33] (Fig. 1). As disease progresses, Aβ has been proposed to activate the Fyn phosphatase, striatal-enriched protein tyrosine phosphatase (STEP), eventually inactivating Fyn, which leads to the loss of synapses and dendritic spine collapse [59, 110] (Fig. 1). Together, these findings demonstrate that Aβ may be the trigger of AD pathogenesis, and tau may be the bullet [9]. From a therapeutic point of view, reducing tau levels or preventing the tau/Fyn or NMDAR/PSD-95 interaction is a suitable strategy to uncouple excitotoxic downstream signaling. Interestingly, the antiepileptic drug Levetiracetam has been shown to reverse synaptic dysfunction, behavioral abnormalities, as well as learning and memory deficits in APP mutant mice [84]. How Levetiracetam exerts its effect is not understood; it has been speculated that it may do so through the following causal chain: suppression of aberrant network activity, reversal of hippocampal remodeling, and recovery of synaptic and cognitive functions. Alternatively, Levetiracetam may block glutamate spillover and thereby block excitotoxic downstream signaling [84].

## Mouse models of AD

AD is a multifactorial disease; the majority of cases are sporadic and age is the greatest risk factor, although genetic and environmental factors can increase a person’s susceptibility. Evidently, no mouse model is capable of fully recapitulating the neuropathological spectrum of the disease [72]. Nonetheless, important aspects of AD have been reproduced in these models, including its biochemical signatures, histopathological hallmarks, behavioral and motor impairments, and, to some degree, neuronal and synaptic loss. This has been assisted by the expression of mutations that are found in FAD and, as far as tau is concerned, in frontotemporal dementia. Interestingly, other than its age of onset, FAD cannot be easily discriminated from cases of late-onset sporadic AD, and the rationale for using mutant transgenic mice has been that, although these mutations are rare, they can nonetheless inform our understanding of the pathogenic process in the more frequent sporadic forms. In FAD, which accounts for less than 1 % of cases, autosomal dominant mutations have been identified in three genes, as mentioned above: *APP*, *PSEN1* and *PSEN2*. The majority of FAD cases are caused by mutations in *PSEN1* and *PSEN2*, and over 130 mutations have been identified. Furthermore, more than 20 pathogenic mutations have been identified in *APP* and several of these, including the V717I ‘London’ mutation [29], the V717F ‘Indiana’ mutation [66], the K670D/M671L ‘Swedish’ mutation [65] and the E693G ‘Arctic’ mutation [69], have been expressed in transgenic mice. In these mice, whether they express mutant *APP* alone or are crossed with mutant *PSEN* mice, elevated Aβ levels are observed at an early age, leading to extracellular amyloid plaque deposition over time; NFTs, however, do not form nor do the models exhibit significant neuronal loss, which makes them incomplete models of the human disease, despite the significant insight they have provided into pathogenic mechanisms.

No familial mutations in *MAPT* have been observed in AD; however, mutations have been identified in patients with FTDP-17 [31]. Several of the known mutations in *MAPT* have been expressed in transgenic mice, including N279 K, ΔK280, P301L, P301S, V337 and R406W, which recapitulate tau aggregation, NFT formation and, to some degree, neuronal loss. For example, transgenic mice expressing P301L mutant tau develop progressive motor and behavioral abnormalities, with robust NFT pathology and neuronal loss in the spinal cord as early as 6.5 months of age [55].

The crosstalk of tau and Aβ was addressed in rats, several years before the first transgenic mouse models with tau and Aβ pathology became available. Stereotaxic injections of Aβ into the rat brain induced both a pronounced immuno-reactivity for ALZ50, a marker of tau pathology, as well as massive neuronal loss [27]. Interestingly, Aβ injections led to tau pathology even at distant sites [91]. With the identification of pathogenic mutations in the *APP* and *MAPT* genes in AD and FTDP-17, respectively, mouse models with more pronounced pathologies became available which were employed to study the interaction of tau and Aβ in more detail. When APP mutant mice were crossed with P301L tau mutants, the double mutants exhibited a substantially enhanced NFT pathology in the limbic system and olfactory cortex compared to the single transgenic P301L tau mice, despite similar levels of tau expression [55]. In contrast, amyloid plaque number, distribution and density did not differ between the double transgenic and the APP mutant mice. It could be argued that the effects on tau elicited by co-expression of mutant APP are, in fact, not due to Aβ but rather to APP or other fragments generated in the process of proteolytic cleavage of APP. However, using a complementary approach and stereotaxically injecting Aβ into a second P301L tau transgenic strain, it was clearly shown that Aβ is capable of causing increased pathological tau phosphorylation and NFT formation [30]. Different from the Aβ injections into P301L tau mice, injection into wild-type mice failed to induce a tau pathology. In a subsequent study, dilute brain extracts from aged APP mutant mice were infused into the hippocampus of young P301L tau transgenic mice. Six months later, tau pathology was observed not only in the hippocampus but also in brain regions to which it is connected, such as the entorhinal cortex and amygdala [12]. Together these findings demonstrate that Aβ can accelerate a pre-existing tau pathology in anatomically separated areas, but that the opposite relationship cannot be established.

In combining the two pathologies into one mouse model, the first triple transgenic model (3xTg-AD) was generated by co-expressing mutant forms of PSEN1, APP and tau, rather than by crossing independent mouse lines [72]. The mice developed extracellular Aβ deposits in the neocortex and hippocampus prior to NFT formation, which was first apparent in the hippocampus, particularly within pyramidal neurons, and later progressed to involve cortical structures. The mice also exhibited deficits in synaptic plasticity, including long-term potentiation, which occurred prior to extracellular Aβ deposition and the formation of NFTs. Interestingly, the deficits in synaptic plasticity correlated with intracellular Aβ-immunogenicity, suggesting a role for intracellular Aβ in neurotoxicity. Another transgenic strain combining mutant APP, PSEN2 and tau (^triple^AD) was analyzed with a particular focus on mitochondrial function, based on proteomic data that were obtained in these mice showing that one-third of deregulated proteins were mitochondrial. The ^triple^AD mice demonstrated that both Aβ and tau act synergistically to induce mitochondrial dysfunction and that this was aggravated in aged mice in the presence of plaques and tangles [80].

## Towards improved models of AD

Most researchers would probably agree that the existing transgenic mouse models have contributed significantly to our understanding of the pathomechanisms of AD, providing useful tools for the validation of drug targets, the development of treatment strategies and biomarkers, and the validation of therapeutic interventions. Yet any model has its limitations, including non-human primates, which, although they are excellent models for normal aging and Aβ neuropathology, lack the tau pathology observed in human disease. A species other than mice that is worth considering, however, is the degu, a social rodent of South-American origin, which has a lifespan of 6–8 years and an Aβ sequence that is identical to that of humans [42, 101]. Unfortunately, the importation of degus can present a hurdle (because they are considered to be a potential pest) and holding costs and requirements are significantly higher than for mice.

What are the alternatives? In the case of mice, the advent of inducible systems has enabled the field to achieve a pathology, be it for tau or for Aβ, that is more robust and develops faster than with conventional transgenic techniques [85, 102]. A second approach is the use of inducible expression systems to achieve brain tissue-specific expression of tau or Aβ. This has proven particularly useful for addressing aspects of the prion-like spreading of pathology along neuronal projections. An example of this is the use of an entorhinal cortex (EC)-specific promoter to drive tau expression alone or with APP expression. In EC-tau mice, age-dependent pathology was observed in the EC after several months, whereas in EC-tau/APP mice, age-dependent pathology was observed not only in the EC, but also in the perirhinal and posterior parietal cortices where the promoter conferring EC-specific expression is supposedly not active. This suggests that APP acts as an accelerant to drive tau toxicity and the spread of pathology observed in AD [47].

Finally, with the advent of gene editing methods such as TALEN and CRISPR it has become much easier to generate knock-in mice and, in particular, to combine several mutations ad libitum in one gene [109]. These studies should lead to a refinement in the analysis and rapid dissection of modifiers of tau and Aβ pathology.

## AD therapeutic strategies

**Fig. 2:**
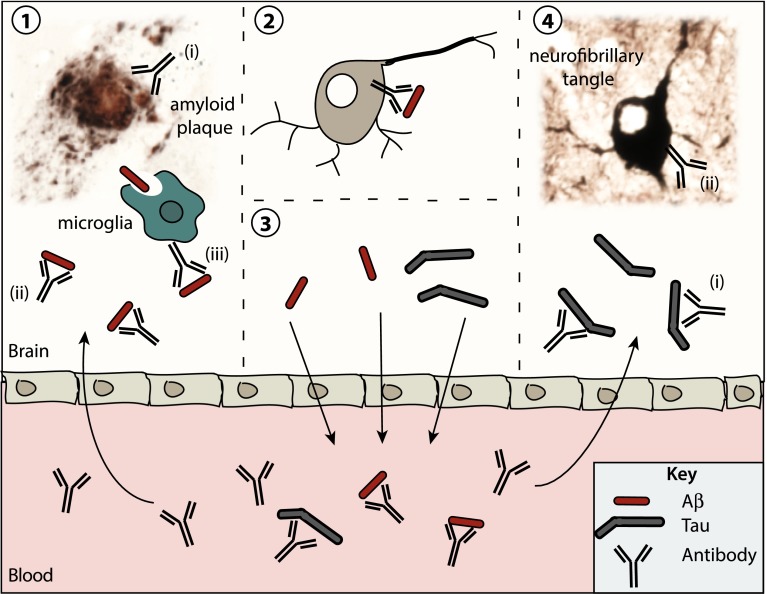
Proposed mechanisms of action of Alzheimer’s disease immunotherapies. AD is characterized pathologically by the aggregation of Aβ and tau and their deposition as extracellular amyloid plaques and intracellular NFTs, respectively. On their pathway to forming plaques and NFTs, Aβ and tau exist as small oligomers that aggregate to fibrils and are the best correlate of neurotoxicity. Preventing Aβ and tau aggregation and promoting their clearance from the brain are the main goals of current immunotherapeutics for the treatment of AD and are depicted in this model. (*1*) Anti-Aβ antibodies that cross the blood–brain barrier (*i*) bind to aggregated Aβ and aid in its disaggregation, (*ii*) bind to soluble Aβ and prevent further Aβ aggregation and/or binding to neurons, and *iii* activate resident microglia to phagocytose soluble Aβ and help dissolve Aβ plaques. (*2*) Endocytosed antibody–target complexes are cleared via the neuronal lysosome. (*3*) ‘Peripheral sink hypothesis’ whereby the antibody-mediated depletion of peripheral Aβ and tau causes an efflux of these proteins from the brain into the blood. (*4*) Anti-tau antibodies in the brain (*i*) bind to secreted tau and prevent its ability to ‘seed’ neighboring neurons or (*ii*) are endocytosed by neurons and bind to intracellular tau. Included in this model is a Campbell–Switzer stained amyloid plaque from a mutant APP transgenic mouse and a Gallyas silver-stained NFT within a neuron of a mutant tau transgenic mouse

Previous attempts to treat AD can be broadly classified into three categories: general symptomatic treatments, those for neuropsychiatric symptoms, and proposed disease-modifying strategies [5]. The first category comprises cholinesterase inhibitors and the NMDAR antagonist memantine, the second anticonvulsants, antidepressants and atypical antipsychotics such as risperidone, and the third ranges from natural products such as vitamin E, gingko biloba and omega-3 fatty acids, to copper and zinc modulators, β- and γ-secretase inhibitors, tau and Aβ aggregation inhibitors and, last but not least, immunotherapies. Several theories exist for the mechanism of action whereby antibodies can clear pathogenic proteins from the brain and reduce the deposition of toxic proteins, particularly as many studies failed to detect antibodies in the brain. The putative mechanisms that have been put forward include (1) inhibition of protein aggregation and ‘seeding’ in the brain, (2) clearance via endosomal/lysosomal degradation after internalization of antibody complexes, (3) microglial receptor-mediated clearance, and (4) establishment of a ‘peripheral sink’ whereby removal of a target protein in the periphery would decrease brain levels by osmosis (Fig. 2). Although this article is not intended to provide a comprehensive review of the currently pursued therapeutic strategies, we will discuss what has been achieved by immunotherapy and, in particular, how the inherent limitations of this approach can possibly be overcome by novel strategies that involve single-chain antibodies.

Anti-Aβ monoclonal antibodies were first demonstrated to dissolve Aβ aggregates in vitro [93] and, since then, both active [88] and passive vaccination [6] have been shown to prevent plaque deposition, reduce the number of pre-established plaques and slow cognitive decline in transgenic mice expressing APP with the V717F mutation. As these transgenic mice do not form NFTs, the ability of Aβ-targeted immunotherapeutics to improve cognition in the presence of hyperphosphorylated tau, and to reduce the progression of other key components of AD neuropathology, could not be investigated. However, administration of anti-Aβ antibodies into the hippocampus of 3xTg-AD mice resulted in a reduction in early tau pathology, which occurred after the clearance of Aβ [71, 73]. Furthermore, decreasing soluble Aβ without affecting soluble tau levels did not improve cognition, suggesting that a reduction in both is required to rescue cognitive impairments in this mouse model [73]. A 35–40 % reduction in tau hyperphosphorylation was also observed in APP Swedish mutant mice with a genetic deletion of nitric oxide synthase 2 (NOS_2_) after active immunization with Aβ_1–__42_ [105]. In P301L tau transgenic mice, however, active immunization with Aβ_1–__42_ was not sufficient to prevent the effect of Aβ on tau aggregation despite the high serum anti-Aβ titer in this model system [48]. Taken together, these studies suggest that anti-Aβ immunotherapeutics may be beneficial for the treatment of AD but not suffice for the treatment of tauopathies in the absence of advert Aβ pathology.

Despite the encouraging results observed with anti-Aβ active and passive immunization in animal models of AD, the first active vaccination trial of patients with mild-to-moderate AD, which used a synthetic Aβ_1–__42_ peptide together with the adjuvant QS-21 (Elan/Wyeth AN1792), was halted when 6 % of immunized patients developed aseptic meningoencephalitis and leukoencephalopathy [68, 74]. Subsequent analysis of the patients that were immunized revealed lower levels of aggregated tau in neuronal processes compared to unimmunized AD cases, but not in the cell bodies where NFTs reside [10].

The side effects observed after active immunization shifted the focus towards passive immunization using humanized anti-Aβ monoclonal antibodies, as this was perceived to be a safer approach. The first passive anti-Aβ antibody to be tested in AD was Bapineuzumab, a humanized N-terminal specific anti-Aβ antibody. Although Bapineuzumab reduced Aβ burden in AD patients in two phase II clinical trials, it recently failed to improve clinical outcomes compared to placebo in phase III trials. Among patients who carry apolipoprotein E allele ε4 (ApoEε4), an allele which increases the risk of developing AD, Bapineuzumab was associated with reduced cerebrospinal fluid (CSF) phospho-tau concentrations and a decreased rate of accumulation of Aβ in the brain [83]. This suggests that Bapineuzumab may be beneficial for asymptomatic ApoEε4 carriers. Similar to Bapineuzumab, the anti-Aβ monoclonal antibody, Solanezumab, which binds to the central region of Aβ, also recently failed to show efficacy in a phase III clinical trial of patients with mild-to-moderate AD. It did, however, slightly slow the rate of cognitive decline in those with mild AD, although this effect did not reach statistical significance. Investigators observed an increase in plasma Aβ levels, suggesting a peripheral sink mechanism of action; however, an increase in total Aβ levels in the CSF was also observed compared to the placebo-treated group. This could be accounted for by the stabilizing effect of the antibody on Aβ as antibody-bound Aβ has a significantly prolonged half-life [54, 89]. Additionally, there was no significant change in the CSF level of tau or phospho-tau in either group [25].

Clinical trials are currently being conducted for the next phase of immunotherapies, which have moved away from linear Aβ epitopes targeting soluble Aβ to those that recognize conformational epitopes on Aβ aggregates; for example SAR228810, Gantenerumab [11], BAN2401, Aducanumab, ACI-24 [64] and Crenezumab [1] (http://www.clinicaltrials.gov). This reflects a growing awareness of the relationship between Aβ oligomers and synaptotoxicity [79]. One example of this approach is Crenezumab, a monoclonal antibody that recognizes multiple forms of aggregated Aβ. Recent Phase II data of Crenezumab demonstrated positive trends in cognitive endpoints over time in patients with mild to moderate AD but failed to show efficacy. Crenezumab is currently being tested in asymptomatic members of a large Colombian family that has a rare *PSEN1* mutation, which predisposes them to AD in middle age. Results from this study will determine if anti-Aβ immunotherapies are more successful when treatment commences prior to the onset of symptoms.

The high specificity and affinity of antibodies for their targets, together with their successful application in the treatment and prevention of non-neurodegenerative diseases, suggest that active or passive immunization remains a promising therapeutic strategy for AD. As the anti-Aβ therapies are failing to meet critical end points in clinical trials, however, one cannot rule out the possibility that tau might be a more effective therapeutic target. Unfortunately, anti-tau immunotherapy studies lag significantly behind those for Aβ. This may reflect the belief that an effective anti-tau therapeutic would be limited to molecules that could cross the plasma membrane. Contrary to this view, however, results from studies investigating tau-specific active and passive immunization in tau transgenic mice have been promising, suggesting a limited level of protection against tau neurotoxicity despite the possible inability of the antibodies to cross the cell membrane.

The initial tau active immunization study, in which wild-type mice were vaccinated with full-length tau, induced the histopathologic features of AD and tauopathies, indicated by the presence of NFTs [82]. However, subsequent active immunization studies with peptides containing phosphorylation epitopes observed in human disease, such as Ser396/404, Ser202/Thr205, Thr212/Ser214, Thr231 and Ser422, produced a significant reduction in tau-related pathology in tau transgenic mice compared to controls [3, 7, 14, 98, 99]. Passive immunization with antibodies that recognize phosphorylated tau has also shown promising results in tau transgenic mice. Immunization with PHF1, a monoclonal antibody that recognizes the phosphorylated residues Ser396/Ser404, resulted in an improvement in behavior on the traverse beam task and a reduction in tau pathology in the dentate gyrus of the hippocampus in tau transgenic mice [13, 21] as well as a significant delay in the onset of motor function decline and weight loss in the P301S tauopathy model [21]. Plasma levels of PHF1 reactivity correlated inversely with tau pathology, indicating that a higher dose of antibodies may have a greater therapeutic effect. Similar results were also obtained after administration of a conformational antibody, MC1, in the same mice [21].

Consistent with the path that studies of anti-Aβ antibodies are taking, the tau field is also focusing on anti-oligomer antibodies, and antibodies which inhibit tau aggregate seeding. TOMA, a tau oligomer-specific monoclonal antibody, was injected once either intracerebroventricularly or intravenously into 8-month-old P301L tau transgenic mice. Just days after the injections, immunized mice exhibited improved cognitive performance and motor activity, and a significant reduction in oligomeric tau levels in the brain and spinal cord [18]. In another study, anti-tau monoclonal antibodies that had been demonstrated to block the seeding activity of P301S tau transgenic brain homogenates in vitro were infused into the lateral ventricle of P301S mice for 3 months. These antibodies significantly reduced hyperphosphorylated, aggregated, and insoluble tau, and improved cognitive defects [108]. It is important to note that, although some of these antibodies have been demonstrated to be internalized by neurons [23, 24, 35], many are unable to do so and therefore function by binding to secreted tau and preventing it from being taken up by neighboring neurons [23]. Furthermore, whereas these antibodies have shown advantageous effects in tau transgenic mice, they have not been tested in transgenic mice exhibiting Aβ pathology and it therefore remains uncertain whether they will be effective strategies for the treatment of AD.

In 2013, phase I clinical trials began to test the efficacy of two active anti-tau vaccines in AD patients. ACI-35 is a synthetic tau peptide which encompasses the Ser396/Ser404 phosphorylation motif [98], whereas AADvac1 targets a truncated, misfolded form of tau [75] and consists of a synthetic peptide derived from a tau protein sequence, which is coupled to the carrier protein keyhole limpet hemocyanin (http://www.clinicaltrials.gov). The results from the ongoing human trials are expected later this year and are eagerly anticipated.

## Future strategies

It has been well demonstrated that Aβ and tau are at the heart of AD pathogenesis and their interplay in the disease is slowly becoming more evident. Theoretically, based on what we know thus far, targeting one of the two molecules in AD should reduce the neurotoxicity of the other. Due to the complexity of AD and the inability to diagnose and treat it early, anti-Aβ immunotherapeutic strategies have generated less than desired results. Therapies targeting tau, or a combination of both anti-Aβ and anti-tau therapies, may be more effective, as it is tau that has been shown to drive Aβ synaptotoxicity. To increase the efficacy of tau immunotherapeutics one might need to consider enhancing their cell-penetrating ability, which could be achieved through the use of antibody engineering.

**Fig. 3:**
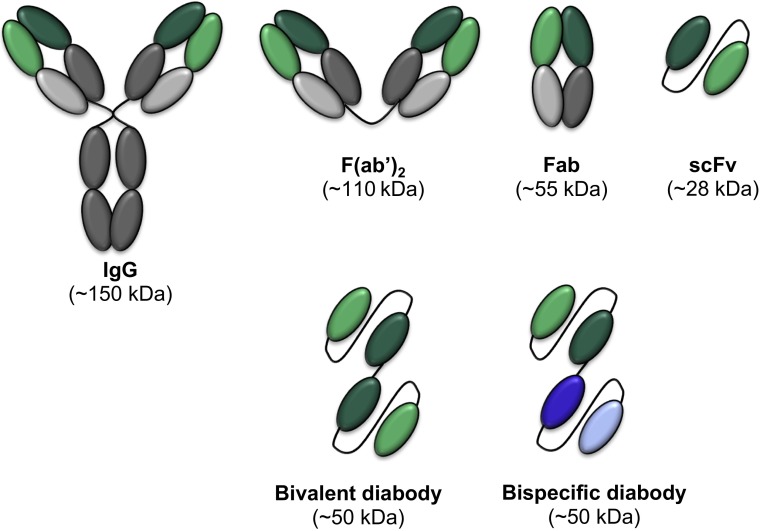
Engineered antibody formats. In addition to full-length ‘classic’ immunoglobulin (IgG) molecules, which are comprised of an antigen-binding fragment (Fab) and a crystallisable fragment (Fc), a variety of antibody variants are currently being exploited for therapeutic intervention. These include: F(ab′)_2,_ which is generated by papain digestion of the whole IgG and lacks the Fc region; Fab, which comprises only one antigen-binding region; scFv, which is composed of the variable heavy and variable light chains of an IgG, joined by a flexible linker; bivalent diabodies, which are composed of two scFvs which have the same target, joined together by a linker; and bispecific diabodies, which are composed of two scFvs with different targets, joined together by a linker

**Fig. 4:**
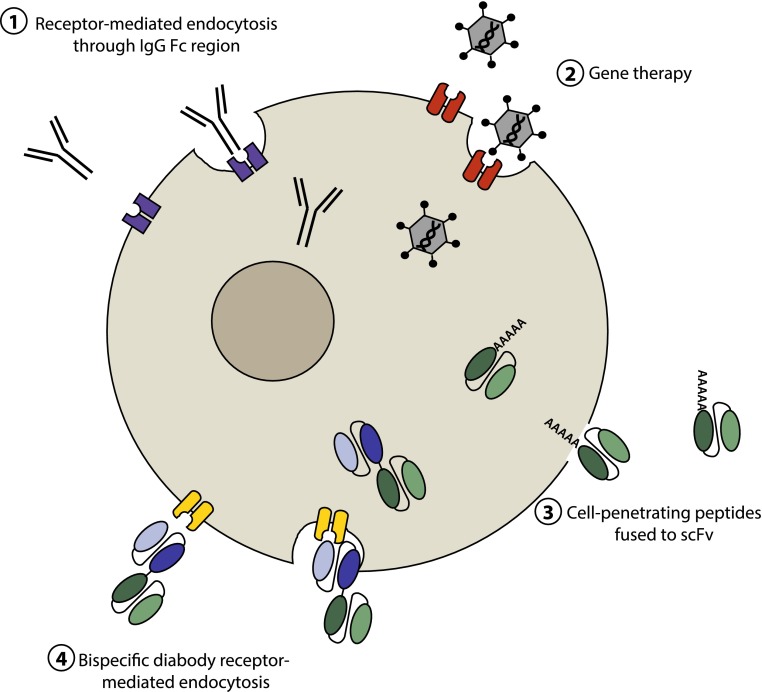
Mechanisms of antibody-mediated cell internalization. The intracellular localization of proteins implicated in disease, such as tau, makes them difficult to target using an immunotherapeutic approach as only very few full-length antibodies are able to penetrate the cell membrane via receptor-mediated endocytosis (*1*). Antibody engineering, however, has generated a number of different avenues one can now pursue to effectively localize functional antibody fragments intracellularly, such as viral-mediated delivery of an scFv gene (*2*); cell membrane penetration via a cell-penetrating peptide fused or conjugated to an scFv (*3*); and receptor-mediated endocytosis of a bispecific scFv with one arm specific for a cell surface receptor (*4*)

Elegant antibody engineering was used to generate a bispecific antibody, which targeted the transferrin receptor and BACE1. Through binding to the transferrin receptor, the bispecific antibody was shown to effectively cross the blood–brain barrier and then bind to BACE1 on the neuronal cell surface, thereby reducing Aβ levels in the brain [111]. This was mediated through the antibody’s low binding affinity to the transferrin receptor as it allowed the antibody to be released following its entry into the brain and thereby enable it to bind to its secondary target, BACE1. Antibody engineering can also be used to generate single-chain variable antibody fragments (scFvs), which are composed of the variable regions of heavy and light immunoglobulin chains joined by a flexible linker; these offer the advantage of expression as a single molecule (Fig. 3). Although relatively underutilized in the neurodegeneration field, scFvs have been extensively used for the diagnosis and treatment of different cancers due to their small size (25 kDa compared to 150 kDa for a full-length antibody) and increased tissue penetration while retaining specificity and high affinity for the target protein [2] (Fig. 3). An additional advantage of scFvs in the context of an AD immunotherapeutic is that they lack the effector function region of the antibody and are therefore unable to induce the complement system, which can lead to harmful side effects such as microhemorrhages or vasogenic edema [77, 78, 106]. Anti-Aβ scFvs have been demonstrated to bind to Aβ with nanomolar affinity, inhibit Aβ aggregation, and prevent Aβ-induced neurotoxicity in vitro [58, 70]. They also reduce Aβ plaques and insoluble Aβ levels in the brains of APP transgenic mice after chronic intranasal delivery [19] As scFvs are single polypeptides they can be further manipulated to include cell-penetrating peptides, allowing the scFv to enter the cells to target intracellular proteins [56] (Fig. 4). They can also be linked together to form bivalent or bispecific diabodies, which exhibit increased stability and binding or dual specificity, respectively [39] (Fig. 3).

Gene therapy delivery of anti-Aβ scFvs using adeno-associated viral (AAV) vectors has demonstrated widespread and long-lasting neuronal delivery and reduced Aβ deposition in APP transgenic mice [53]. Furthermore, gene therapy delivery of an anti-Huntingtin scFv in a transgenic mouse model of Hungington’s disease, using an AAV vector that specifically targets neurons, strongly reduced neuropathology and improved motor and cognitive deficits compared to control [95] (Fig. 4). Genetic fusion of a proteasome-targeting sequence to the scFv was also shown to effectively clear scFv-bound target proteins via the proteasomal pathways, thereby reducing neurotoxic proteins from the cell [17]. These studies suggest that intracellular expression of scFvs (intrabodies) is an effective mechanism for targeting neurodegenerative disease-associated proteins that are predominantly localized within neurons, without stimulating potentially dangerous side effects such as chronic inflammation (Fig. 4).

Lastly, complex diseases, such as HIV, often require the use of a combination therapy approach, which may also be necessary for the treatment of AD. This was tested recently with a phase 1 BACE inhibitor and its phase III antibody which, when given together, reduced Aβ levels and plaque burden more strongly in a transgenic mouse model overexpressing APP, than either treatment did alone [45]. Results such as these might be further improved when multiple proteins, such as Aβ and tau, and multiple neurotoxic pathways are concurrently targeted. Existing therapeutics for the treatment of other diseases or insults characterized by excitotoxicity, such as multiple sclerosis, epilepsy and stroke, could also be used in combination with AD-specific therapies.

## Conclusion

Recent studies have demonstrated a strong interplay between soluble Aβ and tau in the AD pathocascade, prior to their deposition as plaques and NFTs, respectively, with tau acting downstream of Aβ. Consistent with this idea, anti-Aβ therapies have demonstrated the ability to reduce tau pathology in animal models of disease, and to some extent in clinical trials. It is too early to determine whether the reverse is true for anti-tau therapies. As we continue to learn more about the molecular mechanisms underlying the pathogenesis of AD, novel therapeutic targets will be identified, which, when targeted as well, might enhance the effectiveness of current tau and Aβ therapeutics.
